# Estrous cycle state-dependent renewal of appetitive behavior recruits unique patterns of *Arc* mRNA in female rats

**DOI:** 10.3389/fnbeh.2023.1210631

**Published:** 2023-07-13

**Authors:** Emily N. Hilz, Laura A. Agee, Donyun Jun, Marie-H. Monfils, Hongjoo J. Lee

**Affiliations:** ^1^Department of Psychology, The University of Texas at Austin, Austin, TX, United States; ^2^Institute for Neuroscience, The University of Texas at Austin, Austin, TX, United States

**Keywords:** Pavlovian conditioning, renewal, appetitive, estrous cycle, females, catFISH, context

## Abstract

**Introduction:**

Renewal is a behavioral phenomenon wherein extinction learning fails to generalize between different contextual environments, thereby representing a significant challenge to extinction-based rehabilitative therapies. Previously, we have shown that renewal of extinguished appetitive behavior differs across the estrous cycle of the female rat. In this experiment that effect is replicated and extended upon to understand how the estrous cycle may modulate contextual representation at the neuronal population level to drive renewal.

**Methods:**

Estrous cycle stage [i.e., proestrus (P, high hormone) or metestrus/diestrus (M/D, low hormone)] was considered during two important learning and behavioral expression windows: at extinction training and during long-term memory (LTM)/renewal testing. Cellular compartment analysis of temporal activity using fluorescence *in situ* hybridization (catFISH) for *Arc* mRNA was conducted after the distinct context-stimulus exposures.

**Results:**

Rats in P during context-dependent extinction training but in a different stage of the estrous cycle during LTM and renewal testing (P-different) were shown to exhibit more renewal of conditioned foodcup (but not conditioned orienting) behavior compared to rats in other estrous cycle groups. Importantly, we discovered this depends on the order of tests. P-different rats showed differential *Arc* mRNA expression in regions of the prefrontal cortex (PFC), amygdala, and hippocampus (HPC). For each case P-different rats had more co-expression (i.e., expression of both nuclear and cytoplasmic) of *Arc* mRNA compared to other groups; specific to the dorsal HPC, P-different rats also had a more robust *Arc* mRNA response to the extinction context exposure.

**Conclusion:**

These data suggest female rats show estrous cycle state-dependent renewal of appetitive behavior, and differences in context and conditioned stimulus representation at the neuronal level may drive this effect.

## Introduction

Learning rewarding information is important for the biological success of an individual; however, some learners become hyper-fixated with rewarding associations and begin engaging in compulsive reward-seeking behavior. This can lead to maladaptive behaviors like overeating or drug-dependency. Extinction can be used to reduce the occurrence of such behaviors; while useful as a treatment tool, extinction learning often occurs in a rehabilitative setting separate from the place where the behavioral association was originally learned. The generalization of extinction learning from the rehabilitative context to the home context can present a therapeutic challenge because cues that are extinguished within the rehabilitative context can regain their reward-associative power when presented in the original acquisition context (Bouton and Peck, 1989; Wing and Shoaib, 2008), leading to renewal of behavior in a phenomenon known as renewal.

Female rats exhibit less renewal compared to males, and this effect is modulated by exogenous estradiol treatment (Anderson and Petrovich, 2015, 2017). Previously, we extended this to show that the rat estrous cycle also modulates renewal in female rats. In rats, the estrous cycle is a 4- or 5-day period wherein female hormones fluctuate predictably throughout the brain and body (for review see Butcher et al., 1974). Rats in proestrus (P, high hormonal stage) during context-based extinction training, but a different stage of the estrous cycle during renewal testing, exhibited enhanced renewal compared to rats trained and tested in other hormonal states (Hilz et al., 2019a). These animals also showed higher levels of the immediate-early gene product c-Fos after renewal in the hippocampus (HPC), paraventricular nucleus of the thalamus (PVT), and amygdala, suggesting increased functional activity in these regions.

The purpose of the current experiment is to replicate and expand upon these previous findings to understand how the estrous cycle modulates renewal of extinguished appetitive behavior. While the circuitry underlying the contextual modulation of conditioned responding is well established in both fear and appetitive conditioning, less is known about how neuronal ensembles in the HPC, prefrontal cortex (PFC), PVT, and amygdala may guide the expression of previously extinguished behaviors (Ji and Maren, 2005, 2007, 2008; Wang et al., 2016; Anderson and Petrovich, 2017, 2018). *Arc* catFISH (cellular compartment analysis of temporal activity using fluorescence *in situ* hybridization) allows examination of neuronal populations selectively activated in response to two temporally distinct events. *Arc* mRNA is an immediate early gene expressed after cell activation that migrates in a predictable pattern from the cell nucleus to the cytoplasm over an established window of time (∼20 min), and the pattern of *Arc* mRNA expression can be used to identify unique and overlapping cell population activity in response to conditioned stimuli (Guzowski et al., 1999; Vazdarjanova et al., 2002; Lee et al., 2016). *Arc* mRNA is expressed in independent populations of neurons in the amygdala and PFC of rats that have undergone context-dependent extinction training, while rats that did not undergo context-dependent extinction training show proportionately greater numbers of cell populations with overlapping *Arc* mRNA expression (Orsini et al., 2013).

In this experiment the selectivity of *Arc* mRNA expression using catFISH after estrous-cycle dependent renewal is examined. Female rats underwent appetitive Pavlovian conditioning, context-specific extinction training, and then were tested for long-term memory (LTM) of extinction training and renewal of appetitive behavior in quick succession (20 min). Stages of the estrous cycle were considered during extinction training and at testing. We then examined *Arc* mRNA expression in regions that support renewal including the PFC, HPC, PVT, and amygdala. We hypothesized that estrous-cycle modulation of renewal in females may occur via better context representation in neuronal populations of the HPC; as contexts are represented by separate neuronal populations in this region, support for this hypothesis would be indicated by less co-expression of *Arc* mRNA in HPC (Wilson and McNaughton, 1993; Vazdarjanova and Guzowski, 2004; Smith and Mizumori, 2006). Alternatively, estrous-cycle modulation of renewal may indicate a failure to retrieve extinction learning in a state-dependent manner via population activity in the PFC and/or amygdala (indicated by less co-expression of *Arc* mRNA in those regions).

## Materials and methods

### Subjects

Forty-one female Sprague-Dawley rats (Envigo, Indianapolis, IN, USA) weighing 200–275 g were used in this study. Rats were pair-housed on a reverse 14:10 light:dark cycle with lights off at 10 AM. Rats had *ad libitum* access to food and water for approximately 1 week after receipt; once acclimated to the colony room, rats received daily vaginal lavage and were food-restricted to reach approximately 90% free-feeding bodyweight for the entirety of the experiment. All procedures were conducted under the approval of the Institutional Animal Care and Use Committee at the University of Texas at Austin and in accordance with NIH guidelines.

### Apparatus

All procedures were conducted using acrylic-aluminum conditioning chambers measuring 30.5 cm W/25.5 cm L/30.5 cm H (Coulbourn Instruments, Whitehall, PA, USA) described in Hilz et al. (2019a). A food-cup apparatus was connected to an external magazine that delivered food-pellets (45 mg TestDiet, Richmond, IN, USA); entries into the food-cup were measured automatically via breaks in an infrared beam. A 2-W bulb served as the conditioned stimulus (CS) that predicted food-pellet delivery. Each chamber was enclosed in a sound- and light-attenuating box (Coulbourn Instruments, Whitehall, PA, USA) which contained a wall-mounted camera used to record conditioning sessions.

### Behavioral procedure

#### Estrous cycle determinations

The estrous cycle stage of each rat was monitored prior to and during experimental procedures using cytological examination of cells collected from the vaginal epithelium via vaginal lavage (40 μl 0.9% saline flush). Samples were collected at the start of each dark cycle and immediately examined under 10× magnification prior to experimental procedures. Each stage of the estrous cycle is associated with specific gonadal hormone levels and can be identified by differing cell structures and concentrations (for review, see Marcondes et al., 2002). We targeted P as our high-hormone estrous cycle stage (identifiable by a high concentration of nucleated epithelial cells) and M/D (sometimes referred to as diestrus 1 and diestrus 2) as stages with comparatively low levels of gonadal hormones (identifiable as a mix of cornified epithelial cells and leukocytes). Rats that did not show regular estrous cycling with at least one proestrus throughout procedures (*n* = 6), or that required more than 3 days to cycle into the appropriate estrous cycle phase for extinction/testing (*n* = 5), were excluded from analyses. This is consistent with observed changes in estrous cycling after mild food restriction, wherein approximately 20% of Sprague-Dawley rats show disruption at 90% bodyweight (Tropp and Markus, 2001).

#### Renewal procedure

**FIGURE 1:**
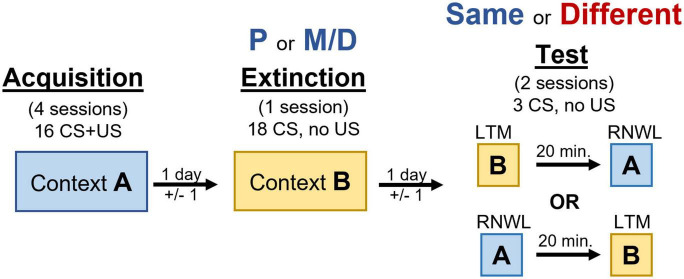
Timeline of behavioral manipulation used in acquisition, extinction, and testing procedures. Renewal (RNWL) and long-term memory for extinction (LTM) tests are counterbalanced and separated by 20 min.

Behavioral procedures like those in Hilz et al. (2019a) were used with slight modifications to allow for the utilization of catFISH for *Arc* mRNA (Figure 1). In short, rats underwent four appetitive conditioning sessions in context A, one extinction training session in context B, and LTM test of extinction recall and renewal of appetitive behavior on the same day separated by 20 min (counterbalanced) in contexts B and A, respectively. The counterbalanced order in which procedures were conducted was ABBA (i.e., acquisition, extinction, LTM, then renewal) or ABAB (i.e., acquisition, extinction, renewal, then LTM). Context A consisted of clear acrylic/aluminum walls, a steel-rod floor, and a neutral scent. Context B consisted of black and white horizontal-lined paper fastened outside the clear acrylic walls, a smooth black floor insert, and clean bedding identical to that used in the home-cage lining the drop-tray beneath the floor to introduce a non-neutral scent.

One day prior to appetitive conditioning rats were trained to retrieve and consume 30 food-pellets delivered on a 60 s fixed interval schedule from the food-cup over a 30-min session. Except for day 1, each appetitive conditioning session consisted of 16 10 s light CS presentations paired with the delivery of a single non-contingent food-pellet over approximately 35 min on a 120 ± 60 s variable interval schedule. The first day of conditioning consisted of 8 consecutive unpaired light CS presentations (no food-pellet) and 8 consecutive light CS presentations paired with the delivery of a single food pellet on the same variable interval schedule. After appetitive conditioning rats underwent one extinction training session consisting of 18 unpaired CS presentations over approximately 40 min on a 120 ± 60 s variable interval schedule. Extinction training typically occurred two days (±1 day) after the final appetitive conditioning session to allow rats to cycle into the appropriate estrous cycle stage. Rats were in either P or M/D during extinction training. Finally, rats were tested for LTM of extinction and renewal of appetitive behavior in two separate testing sessions separated by 20 min on the same day. Each test consisted of 3 unpaired light CS presentations on a 120 ± 60 s variable interval schedule lasting approximately 7 min in contexts A and B. The order these tests were presented was counterbalanced. Rats were categorized as either “same” (i.e., in the same estrous cycle stage as extinction) or “different” (i.e., in a different estrous cycle stage from extinction) during testing. This produced four experimental groups: P-same, P-different, M/D-same, and M/D-different. Additionally, four home-cage control rats in either P or M/D (*n* = 2 per cycle stage, 4 total) with no LTM or renewal testing were used for baseline FISH comparison.

#### Scoring conditioned behavior

Appetitive conditioning was assessed using methods identical to Hilz et al. (2019a). In short, foodcup behavior was measured automatically via breaks in an infrared beam at the opening of the foodcup. Orienting responses were video recorded and scored by an independent observer. Both responses were analyzed over a 15 s interval: the 5 s before the light CS illumination (preCS) and in two blocks of 5 s during the CS illumination (CS1 and CS2). Typically, orienting scores are higher in CS1 and foodcup behavior is higher CS2 (Olshavsky et al., 2014; Hilz et al., 2019b). Therefore, orienting responses from CS1 and foodcup behavior from CS2 were analyzed; these scores were adjusted for baseline.

### Histology

Immediately following the final behavioral test rats were anesthetized with a 0.25 ml pentobarbital and phenytoin solution (Med-Pharmex Inc., Pomona, CA, USA) intraperitoneal injection and transcardially perfused with 0.9% saline followed by 4% paraformaldehyde in 0.1 M phosphate buffer. Brains rested for 24 h at 4°C in paraformaldehyde with 20% sucrose solution prior to rapid freezing with dry ice and storage at −80°C. Brains were sliced into 35 μm coronal sections using a microtome and collected into six adjacent series; each series was mounted on Superfrost Plus microscope slides (Fisher Scientific, Pittsburg, PA, USA), dehydrated overnight in a vacuum chamber, and stored at −80°C in sealed 3 × 1 inch microscope slide boxes (Fischer Scientific).

### Fluorescence *in situ* hybridization

All FISH procedures occurred in an RNAse free environment using procedures modified from Lee et al. (2016), Agee et al. (2023). One full brain series from each rat containing the mPFC, amygdala, dHPC, and vHPC was processed; two brains were excluded due to tissue damage. The plasmid used for generating the *Arc* antisense riboprobe contained the full-length cDNA (∼3.0 kbp) of *Arc* transcript. DNA was cut with 10× digestion buffer (NEBuffer; Biolabs, Ipswich, MA, USA) and 10× *Eco*RI restriction enzyme (Biolabs) in nuclease free water (Ambion). Following purification in EtOH, the DNA pellet was centrifuged, washed in EtOH, and resuspended in a TE buffer. The cRNA probe was made using T7 RNA polymerase (Ambion, Grand Island, NY, USA) and digoxigenin-UTP (DIG RNA labeling mix; Roche Applied Science, Indianapolis, IN, USA). The riboprobe was purified with EtOH precipitation and mini Quick Spin Columns (Roche) then stored at −80°C.

Slides were submerged in 4% paraformaldehyde in 0.1 M phosphate buffer for 1 h to encourage tissue stability. After rinsing in PBS, slides were pretreated with proteinase K and were dehydrated through a series of ascending ethanol dips ranging from 50 to 100% EtOH. Tissue sections were air-dried and coated with ∼300 μl hybridization solution containing the cRNA probe. Slides were temporarily coverslipped and incubated with hybridization solution for ∼20 h at 60°C. After hybridization, coverslips were removed and slides were gently washed in 4X SSC at 60°C before being treated with RNase at 30°C and then washed in descending concentrations of SSC ranging from 4X to 0.1X at 60°C. Slides underwent immunocytochemical processing in a humid chamber using the PerkinElmer TSA Fluorescein system (NEL701001KT; PerkinElmer, Waltham, MA, USA). Tissues were coated with blocking buffer for 30 min prior to anti-DIG-HRP conjugate for 2 h. Slides were gentle washed and then coated with fluorescein tyramide reagent (FITC) for 30 min in a dark humid chamber. Finally, each slide was coverslipped using a mounting medium that contained the nuclear stain 4′,6-diamidino-2-phenylindole (DAPI) (Vectashield; Vector Lab, Burlingame, CA, USA) and stored at −20°C until image acquisition occurred.

### Image acquisition and analysis

Images were acquired using an Axio Scope A1 microscope (Zeiss, Thornwood, NY, USA) from a subset of rats (*n* = 4 per group, run order ABBA). These animals were chosen for FISH analyses because appetitive behavior was higher in this run order (but not in ABAB) and the rats were randomly chosen to match our smallest sample size group. Regions of interest (i.e., the mPFC, nuclei of the amygdala, dHPC, and vHPC) were identified with nuclear DAPI staining under a 10× objective using Swanson’s Atlas (2004). Once identified, both FITC and DAPI images were taken under 40× objective and these images were compiled using a custom macro script with ImageJ (NIH, Bethesda, MD, USA) software. Because the microscope used was not confocal, samples were collected from a single plane at various levels for each region of interest like in Agee et al. (2023). Specifically, one sample for each coordinate was taken from two subregions of the mPFC: the prelimbic (PL; +4.20, +3.60, and +3.20 mm from Bregma) and infralimbic (IL; +3.20 and +2.80 mm) cortices. One sample was taken from each coordinate of four nuclei of the amygdala: the basolateral nucleus (BLA; −2.00, −2.45, −2.85, and −3.25 mm), the lateral nucleus (LA; −2.45, −2.85, and −3.25 mm), the medial portion of the central nucleus of the amygdala (CeA) (mCeA; −1.53 and −1.78 mm), and the lateral portion of the CeA (lCeA; −1.78 and −2.00 mm). One sample for each coordinate was taken from two subregions of the PVT: the anterior PVT (i.e., PVTa; −1.08, −1.33, −1.53, −1.78, and −2.00 mm) and posterior PVT (i.e., PVTp; −2.85, −3.25, and −3.70 mm). Two samples for each coordinate were taken from two regions of the dHPC: dCA1 and dCA3 (2.45, −2.85, and −3.25 mm). Two samples for each coordinate were taken from two subregions of the vHPC: vCA1 and vCA3 (−4.45, −4.60, −5.00, and −5.25 mm).

**FIGURE 2:**
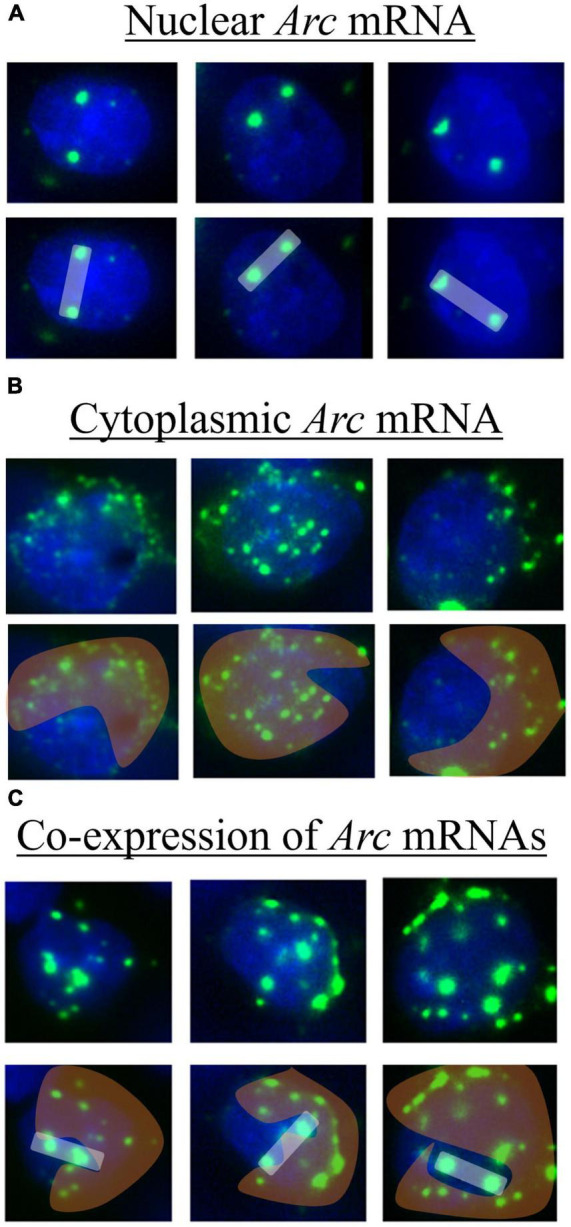
Example of tissue imaged after catFISH procedures showing DAPI stained cells (blue) with various expression patterns of *Arc* mRNA (green). For each, the first row shows the raw image and the second-row highlights expression patterns of nuclear (white bar), cytoplasmic (orange cloud), and co-expression (white bar with orange cloud) of *Arc* mRNA. These images were collected from regions of the mPFC. **(A)** Nuclear *Arc* mRNA expresses as two discrete puncta within a nucleus and correlates temporally to the acquisition context exposure. **(B)** The mRNAs disperse into the cytoplasm in and around the nucleus and correlates temporally to the extinction context exposure. **(C)** Both nuclear and cytoplasmic puncta are present, suggesting the cell is activated by both the context exposures.

Image analysis was conducted by a blind observer using the cell counter plugin with ImageJ software. In the brain, *Arc* mRNA expresses initially within the nucleus as two discrete puncta that we term “nuclear” *Arc* mRNA expression (Figure 2A). Over a short time-course of 20 min, *Arc* mRNA migrates out of the nucleus and disperses into the cytoplasm, which can be inferred by a “cloud” of many puncta around the DAPI-stained nucleus that we term “cytoplasmic” *Arc* mRNA expression (Figure 2B). When the same cell is activated ∼20 min after the first activation, two discrete *Arc* mRNA puncta will again form in the nucleus in addition to the extant cloud of diffused mRNAs in the cytoplasm (Figure 2C). To quantify this, the total number of DAPI-stained cells present in an image were counted; next, DAPI-stained cells containing two clear *Arc* puncta were counted and classified as nuclear *Arc* expression. Then DAPI-stained cells containing diffuse perinuclear *Arc* staining were counted and classified as “cytoplasmic” *Arc* expression. Finally, DAPI-stained cells containing two clear puncta in the nucleus and a diffuse cloud of smaller puncta in and around the nucleus were counted and classified as co-expression of *Arc* mRNA. The number of double cells was then subtracted from the number of nuclear and cytoplasmic cells; this produced a final product of nuclear only, cytoplasmic only, and double only *Arc* expression; these *Arc*+ cells were expressed as a percentage of total DAPI-stained cells and then averaged across samples for subsequent statistical analyses.

### Statistical analyses

All statistical analyses were conducted in R (R Core Team, 2014).

#### Behavior

Appetitive conditioned foodcup and orienting behavior over conditioning, extinction, LTM and renewal testing was analyzed using repeated measures factorial ANOVA. Factors differed depending on the condition analyzed (see section “Results”), and effect size measured as partial eta-squared (η^2^*p*) was provided for significant ANOVAs. Bonferroni corrected *t*-tests were used to analyze *post hoc* comparisons on significant ANOVAs. A measure of effect size, Hedges’ *g* (for unequal sample size) or Cohen’s *d* (for equal sample size), was provided for significant *post hoc* tests for these and all subsequent analyses. Responding during each of the CS presentation periods was also compared for acquisition, extinction, and testing (Supplementary Figure 1).

#### *Arc* mRNA expression

*Arc* mRNA analyses were conducted on experimental rats in the “ABBA” experimental condition (*n* = 4 per group). The percentage of DAPI cells expressing either cytoplasmic (CYT), nuclear (NUC), or co-expressing (DBL) cytoplasmic and nuclear *Arc* mRNA in each region of interest was analyzed using separate between-subjects’ factorial ANOVAs with the factor Group (i.e., Control, P-different, P-same, M/D-different, M/D-same); when a significant difference was detected *post hoc* comparisons were analyzed using Tukey’s HSD. The factors Cycle and Status were compressed into Group to appropriately compare experimental rats to control rats that did not have similar cycle-status designations. The pattern of *Arc* mRNA expression for PVT was not significantly different between groups (Supplementary Figure 2).

#### Context representation

To determine which, if any, of the examined regions might respond more or less to the different context exposures repeated measures ANOVA was used to compare Expression Type (i.e., cytoplasmic, nuclear, or co-expression) between experimental groups (i.e., using Cycle and Status as factors). Control rats did not have separate context exposure and were therefore excluded from this analysis. Bonferroni corrected *t*-tests were used to analyze *post hoc* comparisons on significant ANOVAs.

## Results

### Conditioned responding

#### Appetitive conditioning and extinction

**FIGURE 3:**
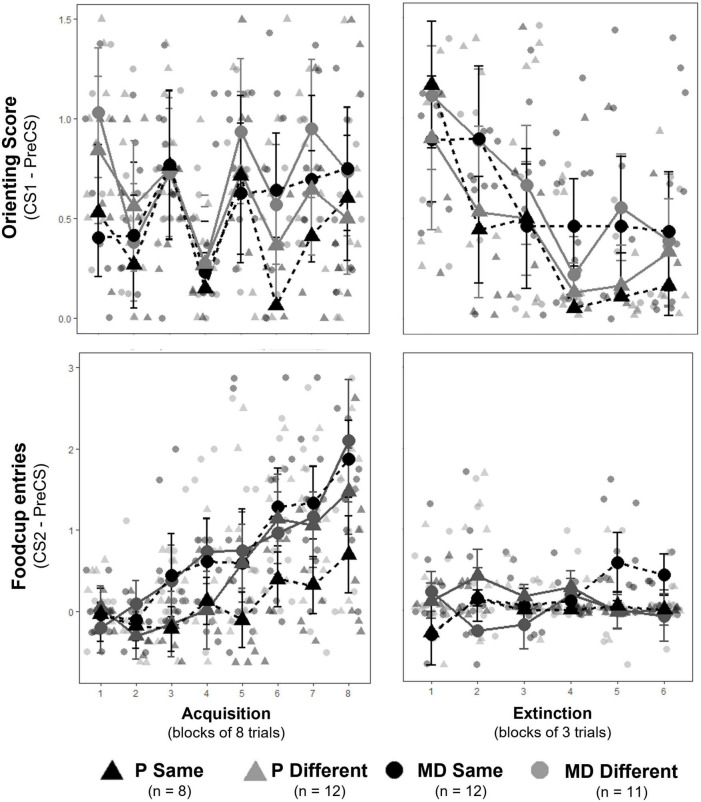
**(Top row)** Orienting responses (OR) ± SEM for acquisition **(left)** and extinction training **(right)**. Individual averages shown behind group scores. Rats acquired ORs over acquisition and extinguished ORs over extinction training similarly between groups. **(Bottom row)** Foodcup (FC) entries ± SEM for acquisition **(left)** and extinction training **(right)**. Individual averages shown behind group scores. Rats significantly acquired FC responses similarly between groups; however, FC behavior was suppressed at the start of extinction and no extinction curve was observed.

The 16 daily appetitive conditioning trials were analyzed in blocks of 8 averaged trials with the exclusion of the unpaired block 1 (when the light CS was presented without the food US) for conditioned foodcup and conditioned orienting behavior. The 18 extinction training trials were analyzed in blocks of 3 averaged trials Factors for both included Training Blocks, Group, and Test Order. *Conditioned orienting*: a significant main effect of Training Blocks indicated all groups acquired conditioned orienting (OR) behavior [*F*(6,180) = 7.00, *p* < 0.001, η^2^*p* = 0.19; Figure 3, top left); there were no effects of Group [*F*(3,30) = 0.68], Test Order [*F*(1,30) = 0.09], nor their interaction [*F*(3,30) = 0.57]. A significant interaction between Group, Block, and Test Order [*F*(6,180) = 2.64, *p* = 0.01, η^2^*p* = 0.08] suggested conditioned orienting differed depending on experimental group and test order. To determine if groups differed at the end of conditioning, a separate 2 × 2 × 2 factorial ANOVA was conducted on the final acquisition block (i.e., block 8). No main effect of Group, Test Order, nor interactions between these variables [*F*(1,30) = 0.46, 0.18, and 0.78, respectively, all *p* > 0.1] indicated all groups had a similar level of OR by the end of conditioning. Subsequently, groups extinguished OR as indicated by a significant main effect of Block [*F*(5,135) = 6.78, *p* < *0.001*, η^2^*p* = 0.20] but no effects of Group [*F*(3,27) = 0.77], Test Order [*F*(1,27) = 0.96], nor interaction between the three factors [*F*(15, 135) = 0.55] indicated groups similarly decreased ORs over extinction blocks (Figure 3, top right). *Conditioned foodcup*: a significant main effect of Training Blocks indicated all groups acquired food-cup (FC) behavior [*F*(6,176) = 19.96, *p* < 0.001, η^2^*p* = 0.40; Figure 3, bottom left]. A main effect of Group was also detected [*F*(1,28) = 3.50, *p* < 0.05, η^2^*p* = 0.27]; *post hoc* analyses indicated P-same rats had less overall FC entries compared to all groups (P different: *p* < 0.05, MD-same and different: *p* < 0.0001) independent of block or test order. However, there was no effect of Test Order [*F*(1,28) = 0.18] nor interaction between the three factors [*F*(18,176) = 1.07]. Groups did not significantly extinguish FC behavior over the extinction training as indicated by no main effect of Block [F(5,160) = 0.90; Figure 3, bottom right], Group [*F*(3,31) = 2.25], Test Order [*F*(1,31) = 0.90], nor interaction of the three factors [*F*(15,160) = 1.41].

#### Renewal

**FIGURE 4:**
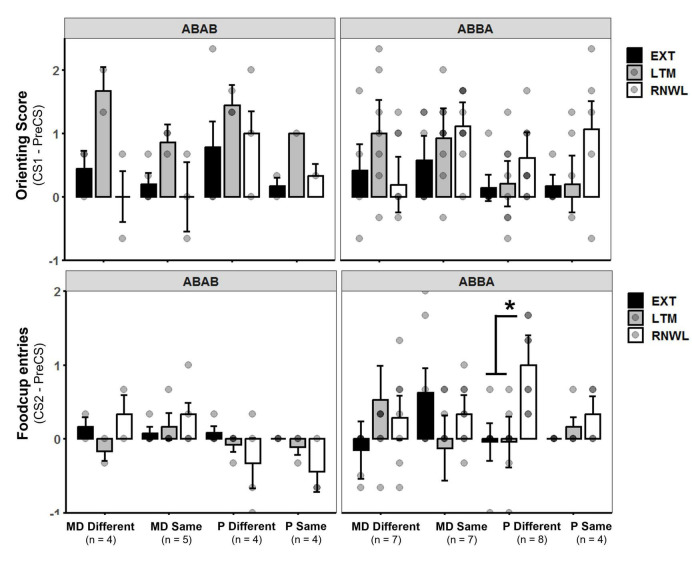
**(Top row)** Orienting response (OR) score ± SEM of last three extinction trials (EXT), long-term memory probe (LTM), and renewal test (RNWL) for ABAB test order **(left)** and ABBA test order **(right)**. Rats showed more OR at LTM compared to EXT between groups. **(Bottom row)** Foodcup entry (FC) score ± SEM of last three extinction trials, LTM probe, and renewal test for ABAB test order **(left)** and ABBA test order **(right)**. P-different rats exhibited significant return of FC behavior during RNWL compared to LTM (*p* < 0.01) in the ABBA test order. *p < 0.05.

The three trials of renewal were compared to the three trials of the LTM probe and the last three trials of extinction for conditioned foodcup and conditioned orienting behavior. Factors included Condition (i.e., EXT, LTM, or Renewal), Group, and Test Order. *Conditioned Orienting*: a significant main effect of Condition [*F*(2,270) = 4.20, *p* = 0.01, η^2^*p* = 0.03] and significant interactions between Condition and Test Order [*F*(2,270) = 2.95, *p* = 0.05, η^2^*p* = 0.02] indicated differences in orienting responses depending on the order of tests. *Post hoc* analyses with an adjusted alpha of *p* < 0.008 indicated orienting responses at LTM were significantly higher than at EXT across all groups (*p* = 0.006, main effect); no other relationships were significant at the adjusted level (Figure 4, top row). *Conditioned foodcup:* a significant main effect of Test Order [*F*(1,31) = 4.34, *p* < 0.05, η^2^*p* = 0.12] was detected along with significant interactions between Group and Condition [*F*(2,303) = 3.05, *p* < 0.05, η^2^*p* = 0.14] and Group, Condition, and Test Order [*F*(2,303) = 4.13, *p* = 0.01, η^2^*p* = 0.18]; a non-significant main effect of Condition was also detected [*F*(2,303) = 2.73, *p* = 0.07]. *Post-hoc* tests with an alpha level of *p* < 0.025 were used to compare conditioned FC behavior for test order. FC behavior was significantly higher in the renewal test when the test order was ABBA compared to ABAB (*p* < 0.01, *g* = 0.59). Because renewal was higher in ABBA, *post-hoc* tests with an alpha level of *p* < 0.0125 were used to compare EXT, LTM, and renewal between experimental groups in the ABBA test order and showed that P-different rats exhibited significantly more renewal of FC behavior (*p* < 0.01, *d* = 0.84) at the renewal test compared to the LTM test (Figure 4, bottom row).

### *Arc* mRNA expression

For each region, the percent of DAPI-stained cells expressing nuclear, cytoplasmic, or co- expressing nuclear and cytoplasmic *Arc* mRNA were first compared between P and M/D home-cage controls using one-way ANOVA; no significant differences in *Arc* mRNA expression were observed based on the estrous cycle. Home-cage controls were combined into a single control group for subsequent analyses (*n* = 4).

#### Prefrontal cortex

**FIGURE 5:**
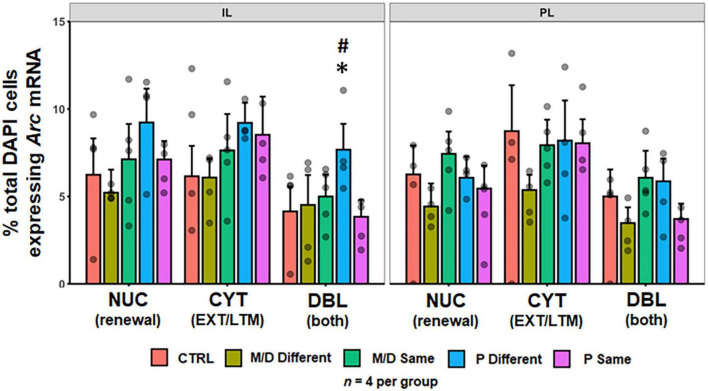
Percent of DAPI+ cells ± SEM expressing cytoplasmic (CYT), double (DBL), or nuclear (NUC) *Arc* mRNA in the infralimbic (IL, **left**) and prelimbic (PL, **right**) cortices of the PFC. P-different rats had a significantly higher percentage of DBL cells in the IL. *p < 0.05, ^#^*p* < 0.1.

No differences were observed in the percentage of DAPI cells expressing either cytoplasmic or nuclear *Arc* mRNA between groups in the PL [*F*(4,64) = 1.70, *p* > 0.1; *F*(4,64) = 2.43, *p* = 0.056] and IL cortices [*F*(4,60) = 2.14, *p* = 0.08; *F*(4,60) = 2.07, *p* = 0.09]. A significant difference in the percentage of total DAPI cells co-expressing both nuclear and cytoplasmic *Arc* mRNA was detected between groups in both the PL [*F*(4,64) = 3.12, *p* < 0.05, η^2^*p* = 0.16] and IL [*F*(4,60) = 3.48, *p* < 0.05, η^2^*p* = 0.19]. *Post hoc* analyses indicated no significant differences between groups in the PL. Significant *post hoc* comparisons were observed in the IL, wherein P-different rats co-expressed a significantly higher percentage of *Arc* mRNA compared to both M/D-different (*p* < 0.05, *d* = 1.11) and P-same (*p* < 0.05, *d* = 1.22) rats; P-different rats also co-expressed a non-significantly higher percentage of *Arc* mRNA compared to control (*p* = 0.08) rats (Figure 5).

#### Amygdala

**FIGURE 6:**
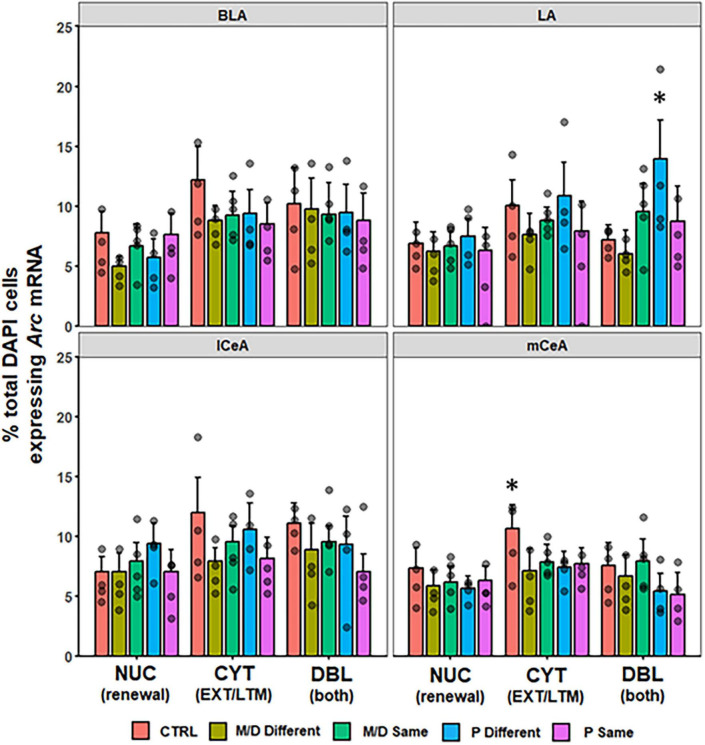
Percent of DAPI+ cells ± SEM expressing CYT, DBL, or NUC *Arc* mRNA in the BLA, LA, lCeA, and mCeA regions of the amygdala. P-different rats had a significantly higher percentage of DBL cells in the LA compared to M/D-different and control (CTRL) rats. CTRL rats had a higher percentage of CYT cells in the mCeA compared to M/D-different and P-same rats in the mCeA. *p < 0.05.

No differences were observed in the percentage of DAPI cells expressing NUC, CYT, or DBL *Arc* mRNA between groups in BLA [*F*(4,82) = 2.14, *p* = 0.08; *F*(4,82) = 1.82, *p* > 0.1; *F*(4,82) = 0.14, *p* > 0.1] nor lCeA [*F*(4,48) = 1.13, *p* > 0.1; *F*(4,48) = 1.70, *p* > 0.1; *F*(4,48) = 1.19, *p* > 0.1]; in the mCeA neither NUC nor DBL were significantly different between groups [*F*(4,81) = 0.84, *p* > 0.1; *F*(4,81) = 2.32, *p* = 0.06]; however, the percentage of total DAPI cells expressing CYT *Arc* mRNA was significantly different between groups [*F*(4,81) = 2.65, *p* < 0.05, η^2^*p* = 0.12]. *Post hoc* analyses indicated control rats expressed a significantly higher percentage of cytoplasmic *Arc* mRNA compared to both M/D- and P-different rats (both *p* < 0.05, *d* = 0.91 and 0.95, respectively) and non-significantly compared M/D-same rats (*p* = 0.08) in mCeA. In LA, neither NUC nor CYT were significantly different between groups [*F*(4,51) = 0.28, *p* > 0.1; *F*(4,51) = 1.18, *p* > 0.1]; however, the percentage of total DAPI cells expressing DBL *Arc* mRNA was significantly different between groups [*F*(4,51) = 4.038, *p* < 0.01, η^2^*p* = 0.24] and *post hoc* comparisons indicated that P-different rats co-expressed a significantly higher percentage of *Arc* mRNA compared to both control (*p* < 0.05, *d* = 1.36) and M/D-different (*p* < 0.01, *d* = 1.46) rats (Figure 6).

#### Hippocampus

**FIGURE 7:**
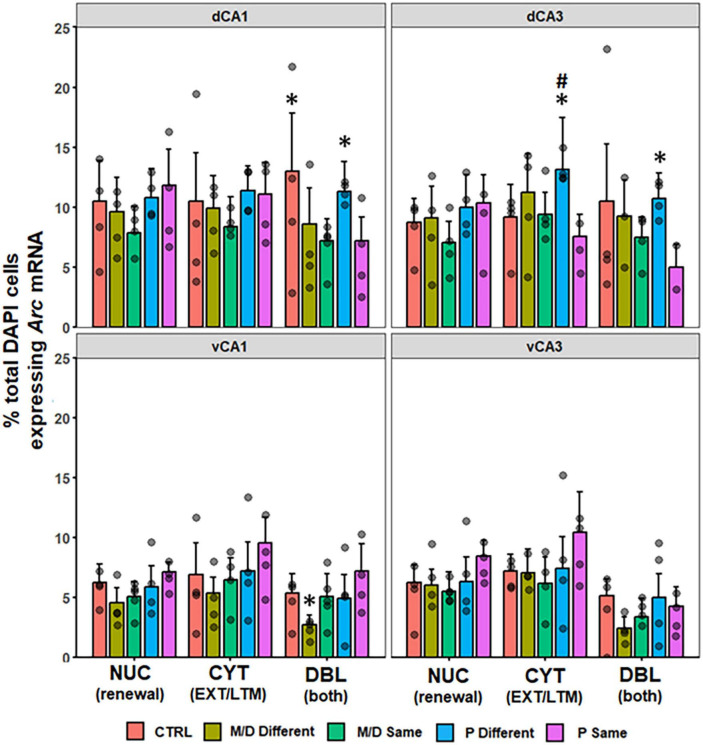
Percent of DAPI+ cells ± SEM expressing CYT, DBL, or NUC *Arc* mRNA in the hippocampus (HPC). In the dorsal HPC (top row), P-different and control (dCA1 only) rats had a significantly higher percentage of DBL cells in both regions compared to M/D- and P-same rats. In dCA3, P-different rats also had significantly more CYT expression compared to P-same and non-significantly to Control rats. *p < 0.05, ^#^*p* < 0.1.

In the dorsal HPC, no differences were observed in the percentage of DAPI cells expressing NUC or CYT *Arc* mRNA between groups in dCA1 [*F*(4,132) = 2.21, *p* = 0.07; *F*(4,132) = 1.38, *p* > 0.1]. In dCA3 there was a significant difference in the percentage of CYT *Arc* mRNA [*F*(4,126) = 3.14, *p* < 0.05, η^2^*p* = 0.09] but not NUC [*F*(4,126) = 2.20, *p* = 0.07] between groups. *Post hoc* analyses indicated P-different rats expressed a significantly higher percentage of cytoplasmic *Arc* mRNA in dCA3 compared to P-same (*p* < 0.05, *d* = 0.84) and non-significantly to control (*p* = 0.08). Both dCA1 and dCA3 showed a significant difference in the percentage of total DAPI cells expressing DBL *Arc* mRNA between groups [*F*(4,132) = 5.17, *p* < 0.001, η^2^ = 0.14; *F*(4,126) = 3.52, *p* < 0.01, η^2^ = 0.10]. In dCA1 (Figure 7, top left), *post hoc* analyses indicated that M/D-same and P-same co-expressed a significantly lower percentage of *Arc* mRNA compared to control (*p* < 0.01, *d* = 0.78; *p* < 0.01, *d* = 0.77) rats, and P-different rats co-expressed a significantly higher percentage of *Arc* mRNA compared to M/D-same rats (*p* < 0.05, *d* = 0.93). In dCA3 (Figure 7, top right), *post hoc* analyses indicated that P-same rats co-expressed a significantly lower percentage of *Arc* mRNA compared to both control (*p* < *0.05*, *d* = 0.75) and P-different (*p* = 0.01, *d* = 1.40) rats (Figure 7). In the ventral HPC, no differences were observed in the percentage of DAPI cells expressing either NUC nor CYT *Arc* mRNA between groups in vCA1 [*F*(4,94) = 2.15, *p* = 0.08; *F*(4,94) = 2.03, *p* = 0.09] and vCA3 [*F*(4,88) = 1.88, *p* > 0.1; *F*(4,88) = 1.93, *p* > 0.1]. The percentage of total DAPI expressing DBL *Arc* mRNA was significantly different between groups in vCA1 [*F*(4,94) = 2.89, *p* < 0.05, η^2^*p* = 0.11] but not vCA3 [*F*(4,88) = 2.18, *p* = 0.07]. *Post hoc* analyses indicated that P-same rats co-expressed a significantly higher percentage of *Arc* mRNA in the vCA1 compared to M/D-different (*p* < 0.01, *d* = 1.33) rats (Figure 7, bottom left).

#### Paraventricular nucleus of the thalamus

The pattern of *Arc* mRNA expression for PVTa and PVTp were not significantly different between groups (Supplementary Figure 2).

#### Context representation

The results of the repeated measures analyses had a similar pattern wherein most regions (i.e., PL, IL, mCeA, LA, BLA, dCA3, vCA1, and vCA3) exhibited significant main effects of Expression Type (statistics detailed in Table 1). Additionally, dCA3 and vCA3 exhibited significant interactions between Group and Expression Type. *Post hoc* analyses with an alpha adjusted to 0.016 for main effects, 0.004 for within-subjects’ interaction terms, and 0.0027 for between-subjects’ interaction terms were conducted to determine the directionality of these effects. Generally, each region with a significant main effect of Expression Type had a higher percentage of DAPI cells expressing CYT *Arc* mRNA compared to NUC and/or DBL of *Arc* mRNA (main effect). Additionally, in the dCA3 P-same rats had a higher percent of NUC and CYT *Arc* mRNA expression than DBL and in vCA3 all experimental groups had a similar relationship of higher CYT than DBL. Finally, in dCA3 M/D-different rats had more nuclear expression of *Arc* mRNA compared to M/D-same rats, and P-different rats had more co-expression than P-same rats.

**TABLE 1:** Within-subjects’ comparison of *Arc* mRNA expression patterns.

Region	Comparison	*F*	*p*	η^2^p	*Post hoc*
**Medial prefrontal cortex**					
PL	Expression type	(2,145) 14.96	<0.001***	0.51	CYT > NUC, *p* = 0.003
					CYT > DBL, *p* < 0.001
	Group *Expression type	(6,145) 1.44	N.S.	–	–
IL	Expression type	(2,142) 13.37	<0.001**	0.42	CYT > DBL, *p* < 0.001
					NUC > DBL, *p* < 0.001
	Group *Expression type	(6,142) 0.48	N.S.	–	–
**Amygdaloid nuclei**					
mCeA	Expression type	(2,199) 5.80	<0.01**	0.26	CYT > DBL, *p* < 0.01 CYT > NUC, *p* < 0.001
	Group *Expression type	(6,199) 1.26	N.S.	–	–
lCeA	Expression type	(2,115) 2.09	N.S.	–	–
LA	Group *Expression type	(6,115) 0.40	N.S.	–	–
	Expression type	(2,121) 7.41	<0.01**	0.4	CYT > NUC, *p* < 0.001
BLA	Group *Expression type	(6,121) 1.59	N.S.	–	–
	Expression type	(2,199) 15.58	<0.001***	0.6	DBL > NUC, *p* < 0.001 CYT > NUC, *p* < 0.001
	Group *Expression type	(6,199) 0.82	N.S.	–	–
**Dorsal and ventral hippocampus**					
dCA1	Expression type	(2,310) 3.12	<0.05*	0.76	CYT > DBL, *p* = 0.06
dCA3	Group *Expression type	(6,310) 1.51	N.S.	–	–
	Expression type	(2,310) 8.35	<0.01**	0.45	CYT > DBL, *p* < 0.001 CYT > NUC, *p* < 0.01
	Group *Expression type	(6,310) 4.52	<0.05*	0.31	P-same: NUC > DBL, *p* = 0.003
					P-same: CYT > DBL, *p* = 0.003
					M/D-different NUC > M/D-same NUC, *p* < 0.001
					P-different DBL > P-same DBL, *p* = 0.001
vCA1	Expression type	(2,22) 10.61	<0.001***	0.49	CYT > DBL, *p* < 0.001
	Group *Expression type	(6,220) 0.53	N.S.	–	–
					CYT > NUC, *p* < 0.001
vCA3	Expression type	(2,202) 32.94	<0.001***	0.75	CYT > DBL, *p* < 0.001 CYT > NUC, *p* < 0.001
					NUC > DBL, *p* < 0.001
	Group *Expression type	(6,202) 4.48	<0.05*	0.29	M/D-same: CYT > DBL, *p* = 0.002
				M/D-different: NUC > DBL, *p* < 0.001
					M/D-different: CYT > DBL, *p* < 0.001
					P-different: CYT > DBL, *p* = 0.002
					P-same: NUC > DBL, *p* < 0.001
					P-same: CYT > DBL, *p* = 0.003

Results list significant main or interaction effects of repeated measures ANOVA for each brain region, alpha (*p*), effect size (η^2^), and post hoc results indicating directionality from Bonferroni corrected *t* tests. *p < 0.05, **p < 0.01, ***p < 0.001.

## Discussion

In this experiment the effect of the estrous cycle on renewal of extinguished appetitive behavior was determined in female rats, and subsequent expression of *Arc* mRNA was examined. Estrous cycle stage (i.e., P or M/D) was considered during two important learning and behavioral expression windows: at extinction training and during LTM/renewal testing. The results of this experiment replicated previous findings with some notable caveats: rats that underwent extinction training in P and LTM/renewal testing in a different stage of the estrous cycle (i.e., P-different rats) exhibited more renewal of appetitive behavior compared to all other experimental groups. Furthermore, this observation was restricted to conditioned foodcup, and not conditioned orienting, behavior. Importantly, this effect was only observed depending on the order testing contexts were presented.

Previous research on renewal of both appetitive and fear behavior suggests the order which test contexts are presented should not influence behavioral expression (Bouton and Ricker, 1994; Rescorla, 2008; Bouton et al., 2011; Todd et al., 2012a,b; Bouton and Schepers, 2015; Trask and Bouton, 2016). The test order effect observed in this experiment is not in accordance with that body of research. The basis for the observed test order effect is difficult to determine, as groups did not differ based on test order in the acquisition or extinction of conditioned foodcup behavior nor was there any reason to believe the rapid test presentations would affect responding because others have successfully used rapid context presentations to examine differences in the expression of extinguished fear behavior (Orsini et al., 2013). The most probable explanation is that small sample sizes associated with the ABAB counterbalance group drove the test order effect, and more systematic exploration of counterbalanced renewal designs could provide illumination although that is beyond the scope of the current work.

As such, the following discussion is constrained to results from the ABBA group that showed behavioral differences based on the estrous cycle. The results of this experiment extend previous findings that neuronal cell activity differs after renewal in a cycle-specific manner. In Hilz et al. (2019a) P-different rats showed more cell activation (measured with c-Fos) in regions of the amygdala and HPC after renewal testing. Here the immediate early gene *Arc* was examined, and differential *Arc* mRNA expression was observed in regions of the amygdala, HPC, and PFC. The pattern of *Arc* mRNA expression was similar to that of FOS in Hilz et al. (2019a) with some notable differences: no differential c-Fos expression was observed in the PFC, but P-different rats did express more c-Fos in the ventral HPC. In this experiment differential expression and co-expression of *Arc* mRNA was observed in the IL region of the PFC, in the dorsal but not the ventral HPC, and in the amygdala. Moreover, it was observed that multiple regions including PL, mCeA, LA, BLA, dCA3, vCA1, and vCA3 were generally more responsive to the LTM context-stimulus exposure than to the renewal context exposure, indicated by overall higher percentage of cytoplasmic than nuclear *Arc* mRNA expression among all groups.

The purpose of this experiment was to understand what may drive estrous-cycle specific differences in renewal both in terms of behavioral and subsequent cellular expression. There were two main hypotheses as to why P-different rats may exhibit renewal compared to other groups: (1) P may enhance extinction context information processing and encoding, and aid in subsequent context-differentiation thereby leading to renewal of behavior after extinction; and/or (2) P acts as a physiological context to which extinction learning becomes state-dependent, and being in a different estrous state leads to renewal of behavior.

Context is represented at a cellular level by separate cell populations in the HPC (Wilson and McNaughton, 1993; Vazdarjanova and Guzowski, 2004; Smith and Mizumori, 2006) and context representations act as a cue to guide conditioned responding in a context-appropriate manner (Balaz et al., 1982; Fanselow, 1990). If P rats were better encoding and differentiating between the LTM and renewal context-stimulus exposures, we would expect to see less co-expression of nuclear and cytoplasmic *Arc* mRNA in the HPC of P rats. It can be inferred that less overlap indicates better or more specific context representation while more overlap would indicate the opposite. In this experiment P-different rats exhibited a higher percentage of cell population overlap in both regions of the dorsal HPC – indicating that the stimulus exposures during the LTM and renewal tests induced *Arc* mRNA expression in the same HPC cell populations. It is notable, however, that P-different rats also exhibited a higher expression cytoplasmic *Arc* mRNA in the dCA3 which is important for rapid context representation (Daumas et al., 2005). Cytoplasmic activity here temporally relates to the extinction/LTM context-stimulus exposure; therefore, P-different rats may have had a better representation of the extinction context compared to others. The lack of a similar pattern of nuclear *Arc* mRNA (temporally related to the acquisition/renewal context-stimulus exposure) in these animals may reflect the lack of state-specificity during the acquisition context learning experience. The increased co-expression in of *Arc* mRNA in P-different rats may indicate cells in the dHPC were responding to the same light CS exposures between contexts, and behavioral expression was gated in some way based on rapid context representation.

A growing body of evidence suggests that estradiol during extinction training, both via the estrous cycle and with exogenous treatment, enhances extinction recall (Chang et al., 2009; Milad et al., 2009, 2010; Zeidan et al., 2011; Graham and Milad, 2013, 2014; Graham and Daher, 2016). The enhancing effects of estradiol on extinction recall may aid in or be representative of a state-dependent effect. Internal states can act as contexts to cue recall of extinguished behavior (Ahlers and Richardson, 1985; Bouton et al., 1990; Eich, 1995; Schepers and Bouton, 2017). P is characterized by dramatic changes in sex steroid hormone level (Butcher et al., 1974; Smith et al., 1975) and likely represents a separate physiological experience from M/D. Therefore, it is possible that the estrous cycle of the female rat may act as an internal context and guide subsequent extinction recall and/or conditioned responding. Rats that undergo extinction training while in P may associate extinction learning and the extinction context with the P stage of the estrous cycle: rats may recall extinction training and subsequently repress or express conditioned responding dependent on the similarity of the internal hormonal context at testing. Support for this hypothesis can be found in recent contextual fear learning research, wherein conditioning and testing in different stages of the estrous cycle affected the level of context freezing behavior in female rats (Blair et al., 2022); that experiment further showed that the effect does not rely on progesterone but does not exclude estradiol. State-dependency of the estrous cycle has also been shown to affect memory for aversive experiences, with female rats showing reduced aversion to quinine when tested in different phases of the cycle from their original exposure (Costanzo et al., 1995). To our knowledge, Hilz et al. (2019a) is the first experiment to show estrous cycle state-dependency of appetitive behavior in context-extinction recall, and this is the first to explore how that state-dependency is represented at the neuronal population level.

Cell populations expressing *Arc* mRNA have less overlap after context-dependent extinction learning in the amygdala and PFC (Orsini et al., 2013), and those regions guide conditioned responding in renewal. We hypothesized that state-dependency would be indicated by P-different rats exhibiting less co-expression of nuclear and cytoplasmic *Arc* mRNA in those regions, because different cell groups would activate in response to the context-stimulus exposures. Again, an opposite result was observed: P-different rats exhibited more overlap of cell population activity in the PFC (specifically in the IL cortex, which is associated with behavioral suppression in extinction) and in the lateral amygdala. This may suggest that for P-different rats, the light CS was activating the same cell populations in these regions at both context exposures in a way not observed in other groups.

It is unclear how *Arc* mRNA co-expression may contribute to behavioral inhibition and expression in a state-dependent manner. The observed overlap of cell population activity may be a facet of differences in *Arc* mRNA recruitment compared to other immediate early genes (e.g., c-Fos). *Arc* mRNA is not induced by stimulus experiences in the same way that FOS is expressed (Kubik et al., 2007); *Arc* mRNA is necessary for neuronal LTP and LTD (Zhang and Bramham, 2021) and is supposed to aid in the creation or strengthening of neuronal connections (Bramham et al., 2010; Nikolaienko et al., 2018); behaviorally, *Arc* is expressed differentially in response to context-dependent cue exposures and mediates memory formation and recall (Korb and Finkbeiner, 2011; Chia and Otto, 2013). Conceptualizing *Arc* as a marker of “new learning” or as necessary for the development of future behavior could alter the interpretation of this experiment: female rats exhibiting renewal may be creating or updating information surrounding the context-stimulus learning and recall experience in overlapping cell populations throughout the renewal network. Because more cytoplasmic *Arc* mRNA was expressed by P-different rats in the HPC, this may also suggest rapid updating or strengthening of the extinction context representation which may have aided substantially in behavioral disinhibition after the context change.

Ultimately, the results of this experiment did not fully elucidate the way hormonal states of the estrous cycle drive renewal in female rats. There is some support for state-dependent extinction context representation and recall, as well as an indication that CS information processing is rapidly updated during renewal. The research has implications for women’s rehabilitative therapy and exemplifies the importance of considering the physiological state of learners as a variable that can affect successful therapeutic outcomes.

## Data availability statement

The raw data supporting the conclusions of this article will be made available by the authors, without undue reservation.

## Ethics statement

The animal study was reviewed and approved by the Institutional Animal Care and Use Committee at University of Texas at Austin.

## Author contributions

EH designed and conducted the experiment, and drafted the manuscript. LA and DJ assisted with tissue processing. M-HM and HL designed and oversaw the experiment, trained EH and LA on catFISH, and approved the final version of the manuscript. All authors contributed to the article and approved the submitted version.

## References

[B1] Agee L. Hilz E. Jun D. Nemchek V. Lee H. Monfils M.-H. (2023). Patterns of Arc mRNA expression in the rat brain following dual recall of fear- and reward-based socially acquired information. *Sci. Rep.* 13:2429. 10.1038/s41598-023-29609-6 36765118 PMC9918527

[B2] Ahlers S. T. Richardson R. (1985). Administration of dexamethasone prior to training blocks ACTH-induced recovery of an extinguished avoidance response. *Behav. Neurosci.* 99 760–764. 10.1037/0735-7044.99.4.760 3040035

[B3] Anderson L. C. Petrovich G. D. (2015). Renewal of conditioned responding to food cues in rats: Sex differences and relevance of estradiol. *Physiol. Behav.* 151 338–344. 10.1016/j.physbeh.2015.07.035 26253218 PMC4587292

[B4] Anderson L. C. Petrovich G. D. (2017). Sex specific recruitment of a medial prefrontal cortex-hippocampal-thalamic system during context-dependent renewal of responding to food cues in rats. *Neurobiol. Learn. Memory* 139 11–21. 10.1016/j.nlm.2016.12.004 27940080 PMC5334368

[B5] Anderson L. C. Petrovich G. D. (2018). Distinct recruitment of the hippocampal, thalamic, and amygdalar neurons projecting to the prelimbic cortex in male and female rats during context-mediated renewal of responding to food cues. *Neurobiol. Learn. Memory* 150 25–35. 10.1016/j.nlm.2018.02.013 29496643 PMC5893354

[B6] Balaz M. A. Capra S. Kasprow W. J. Miller R. R. (1982). Latent inhibition of the conditioning context: Further evidence of contextual potentiation of retrieval in the absence of appreciable context-US associations. *Anim. Learn. Behav.* 10 242–248. 10.3758/BF03212277

[B7] Blair R. S. Acca G. M. Tsao B. Stevens N. Maren S. Nagaya N. (2022). Estrous cycle contributes to state-dependent contextual fear in female rats. *Psychoneuroendocrinology* 141:105776. 10.1016/j.psyneuen.2022.105776 35489312

[B8] Bouton M. Kenney F. Rosengard C. (1990). State-Dependent Fear Extinction With Two Benzodiazepine Tranquilizers. *Behav. Neurosci.* 104 44–55. 10.1037//0735-7044.104.1.44 2317285

[B9] Bouton M. E. Peck C. A. (1989). Context effects on conditioning, extinction, and reinstatement in an appetitive conditioning preparation. *Anim. Learn. Behav.* 17 188–198. 10.3758/BF03207634

[B10] Bouton M. E. Ricker S. T. (1994). Renewal of extinguished responding in a second context. *Anim. Learn. Behav.* 22 317–324. 10.3758/BF03209840

[B11] Bouton M. E. Schepers S. T. (2015). Renewal after the punishment of free operant behavior. *J. Exp. Psychol.* 41 81–90. 10.1037/xan0000051 25706548 PMC4339226

[B12] Bouton M. E. Todd T. P. Vurbic D. Winterbauer N. E. (2011). Renewal after the extinction of free operant behavior. *Learn. Behav.* 39 57–67. 10.3758/s13420-011-0018-6 21279496 PMC3059840

[B13] Bramham C. R. Alme M. N. Bittins M. Kuipers S. D. Nair R. R. Pai B. (2010). The Arc of synaptic memory. *Exp. Brain Res.* 200 125–140. 10.1007/s00221-009-1959-2 19690847 PMC2803749

[B14] Butcher R. L. Collins W. E. Fugo N. W. (1974). Plasma Concentration of LH, FSH, Prolactin, Progesterone and Estradiol-17/3 Throughout the 4-Day Estrous Cycle of the Rat. *Endocrinology* 94:5. 10.1210/endo-94-6-1704 4857496

[B15] Chang Y.-J. Yang C.-H. Liang Y.-C. Yeh C.-M. Huang C.-C. Hsu K.-S. (2009). Estrogen modulates sexually dimorphic contextual fear extinction in rats through estrogen receptor β. *Hippocampus* 19 1142–1150. 10.1002/hipo.20581 19338017

[B16] Chia C. Otto T. (2013). Hippocampal Arc (Arg3.1) expression is induced by memory recall and required for memory reconsolidation in trace fear conditioning. *Neurobiol. Learn. Memory* 106 48–55. 10.1016/j.nlm.2013.06.021 23872190

[B17] Costanzo D. J. Riccio D. C. Kissinger S. (1995). State-dependent retention produced with estrus in rats. *Physiol. Behav.* 57 1009–1011. 10.1016/0031-9384(94)00007-R 7610126

[B18] Daumas S. Halley H. Francés B. Lassalle J.-M. (2005). Encoding, consolidation, and retrieval of contextual memory: Differential involvement of dorsal CA3 and CA1 hippocampal subregions. *Learn. Memory* 12 375–382. 10.1101/lm.81905 16027176 PMC1183255

[B19] Eich E. (1995). Searching for Mood Dependent Memory. *Psychol. Sci.* 6 67–75. 10.1111/j.1467-9280.1995.tb00309.x

[B20] Fanselow M. S. (1990). Factors governing one-trial contextual conditioning. *Anim. Learn. Behav.* 18 264–270. 10.3758/BF03205285

[B21] Graham B. M. Daher M. (2016). Estradiol and Progesterone have Opposing Roles in the Regulation of Fear Extinction in Female Rats. *Neuropsychopharmacology* 41:3. 10.1038/npp.2015.202 26156559 PMC4707823

[B22] Graham B. M. Milad M. R. (2013). Blockade of Estrogen by Hormonal Contraceptives Impairs Fear Extinction in Female Rats and Women. *Biol. Psychiatry* 73 371–378. 10.1016/j.biopsych.2012.09.018 23158459 PMC3557577

[B23] Graham B. M. Milad M. R. (2014). Inhibition of estradiol synthesis impairs fear extinction in male rats. *Learn. Memory* 21 347–350. 10.1101/lm.034926.114 24939838 PMC4061425

[B24] Guzowski J. F. McNaughton B. L. Barnes C. A. Worley P. F. (1999). Environment-specific expression of the immediate-early gene Arc in hippocampal neuronal ensembles. *Nat. Neurosci.* 2:12. 10.1038/16046 10570490

[B25] Hilz E. N. Smith R. W. Hong Y. J. Monfils M. H. Lee H. J. (2019a). Mapping the estrous cycle to context-specific extinction memory. *Behav. Neurosci.* 133 614–623. 10.1037/bne0000343 31599608

[B26] Hilz E. N. Lewis S. M. Park S. Monfils M. H. Lee H. J. (2019b). Extinction to amphetamine-associated context in female rats is dependent upon conditioned orienting. *Psychopharmacology* 236 507–515. 10.1007/s00213-018-5073-7 30343363

[B27] Ji J. Maren S. (2005). Electrolytic lesions of the dorsal hippocampus disrupt renewal of conditional fear after extinction. *Learn. Memory* 12 270–276. 10.1101/lm.91705 15930505 PMC1142455

[B28] Ji J. Maren S. (2007). Hippocampal involvement in contextual modulation of fear extinction. *Hippocampus* 17 749–758. 10.1002/hipo.20331 17604353

[B29] Ji J. Maren S. (2008). Differential roles for hippocampal areas CA1 and CA3 in the contextual encoding and retrieval of extinguished fear. *Learn. Memory* 15 244–251. 10.1101/lm.794808 18391185 PMC2327266

[B30] Korb E. Finkbeiner S. (2011). Arc in synaptic plasticity: From gene to behavior. *Trends Neurosci.* 34 591–598. 10.1016/j.tins.2011.08.007 21963089 PMC3207967

[B31] Kubik S. Miyashita T. Guzowski J. F. (2007). Using immediate-early genes to map hippocampal subregional functions. *Learn. Memory* 14 758–770. 10.1101/lm.698107 18007019

[B32] Lee H. J. Haberman R. P. Roquet R. F. Monfils M.-H. (2016). Extinction and Retrieval + Extinction of Conditioned Fear Differentially Activate Medial Prefrontal Cortex and Amygdala in Rats. *Front. Behav. Neurosci.* 9:369. 10.3389/fnbeh.2015.00369 26834596 PMC4722140

[B33] Marcondes F. K. Bianchi F. J. Tanno A. P. (2002). Determination of the estrous cycle phases of rats: Some helpful considerations. *Brazil. J. Biol.* 62 609–614. 10.1590/S1519-69842002000400008 12659010

[B34] Milad M. R. Igoe S. A. Lebron-Milad K. Novales J. E. (2009). Estrous cycle phase and gonadal hormones influence conditioned fear extinction. *Neuroscience* 164 887–895. 10.1016/j.neuroscience.2009.09.011 19761818 PMC2783784

[B35] Milad M. R. Zeidan M. A. Contero A. Pitman R. K. Klibanski A. Rauch S. L. (2010). The influence of gonadal hormones on conditioned fear extinction in healthy humans. *Neuroscience* 168 652–658. 10.1016/j.neuroscience.2010.04.030 20412837 PMC2881679

[B36] Nikolaienko O. Patil S. Eriksen M. S. Bramham C. R. (2018). Arc protein: A flexible hub for synaptic plasticity and cognition. *Semin. Cell Dev. Biol.* 77 33–42. 10.1016/j.semcdb.2017.09.006 28890419

[B37] Olshavsky M. E. Shumake J. Rosenthal A. A. Kaddour-Djebbar A. Gonzalez-Lima F. Setlow B. (2014). Impulsivity, risk-taking, and distractibility in rats exhibiting robust conditioned orienting behaviors. *J. Exp. Anal. Behav.* 102 162–178. 10.1002/jeab.104 25130520

[B38] Orsini C. A. Yan C. Maren S. (2013). Ensemble coding of context-dependent fear memory in the amygdala. *Front. Behav. Neurosci.* 7:199. 10.3389/fnbeh.2013.00199 24379767 PMC3861741

[B39] R Core Team (2014). *R: A language and environment for statistical computing.* Vienna: R Foundation for Statistical Computing. Available online at: https://www.R-project.org/

[B40] Rescorla R. A. (2008). Within-Subject Renewal in Sign Tracking. *Q. J. Exp. Psychol.* 61 1793–1802. 10.1080/17470210701790099 18609366

[B41] Schepers S. T. Bouton M. E. (2017). Hunger as a Context: Food Seeking That Is Inhibited During Hunger Can Renew in the Context of Satiety. *Psychol. Sci.* 28 1640–1648. 10.1177/0956797617719084 28957015 PMC5673576

[B42] Smith D. M. Mizumori S. J. Y. (2006). Hippocampal place cells, context, and episodic memory. *Hippocampus* 16 716–729. 10.1002/hipo.20208 16897724

[B43] Smith M. S. Freeman M. E. Neill J. D. (1975). The Control of Progesterone Secretion During the Estrous Cycle and Early Pseudopregnancy in the Rat: Prolactin, Gonadotropin and Steroid Levels Associated with Rescue of the Corpus Luteum of Pseudopregnancy ^1^ ^2^. *Endocrinology* 96 219–226. 10.1210/endo-96-1-219 1167352

[B44] Todd T. P. Winterbauer N. E. Bouton M. E. (2012a). Contextual control of appetite. Renewal of inhibited food-seeking behavior in sated rats after extinction. *Appetite* 58 484–489. 10.1016/j.appet.2011.12.006 22200411 PMC3579161

[B45] Todd T. P. Winterbauer N. E. Bouton M. E. (2012b). Effects of the amount of acquisition and contextual generalization on the renewal of instrumental behavior after extinction. *Learn. Behav.* 40 145–157. 10.3758/s13420-011-0051-5 22002545

[B46] Trask S. Bouton M. E. (2016). Discriminative properties of the reinforcer can be used to attenuate the renewal of extinguished operant behavior. *Learn. Behav.* 44 151–161. 10.3758/s13420-015-0195-9 26400498 PMC4805475

[B47] Tropp J. Markus E. J. (2001). Effects of mild food deprivation on the estrous cycle of rats. *Physiol. Behav.* 73 553–559. 10.1016/S0031-9384(01)00487-5 11495659

[B48] Vazdarjanova A. Guzowski J. F. (2004). Differences in Hippocampal Neuronal Population Responses to Modifications of an Environmental Context: Evidence for Distinct, Yet Complementary, Functions of CA3 and CA1 Ensembles. *J. Neurosci.* 24 6489–6496. 15269259 10.1523/JNEUROSCI.0350-04.2004 PMC6729865

[B49] Vazdarjanova A. McNaughton B. L. Barnes C. A. Worley P. F. Guzowski J. F. (2002). Experience-Dependent Coincident Expression of the Effector Immediate-Early Genes Arc and Homer 1a in Hippocampal and Neocortical Neuronal Networks. *J. Neurosci.* 22 10067–10071. 12451105 10.1523/JNEUROSCI.22-23-10067.2002 PMC6758761

[B50] Wang Q. Jin J. Maren S. (2016). Renewal of extinguished fear activates ventral hippocampal neurons projecting to the prelimbic and infralimbic cortices in rats. *Neurobiol. Learn. Memory* 134 38–43. 10.1016/j.nlm.2016.04.002 27060752 PMC5018421

[B51] Wilson M. A. McNaughton B. L. (1993). Dynamics of the hippocampal ensemble code for space. *Science* 261 1055–1058. 10.1126/science.8351520 8351520

[B52] Wing V. C. Shoaib M. (2008). Contextual stimuli modulate extinction and reinstatement in rodents self-administering intravenous nicotine. *Psychopharmacology* 200 357–365. 10.1007/s00213-008-1211-y 18587561

[B53] Zeidan M. A. Igoe S. A. Linnman C. Vitalo A. Levine J. B. Klibanski A. (2011). Estradiol Modulates Medial Prefrontal Cortex and Amygdala Activity During Fear Extinction in Women and Female Rats. *Biol. Psychiatry* 70 920–927. 10.1016/j.biopsych.2011.05.016 21762880 PMC3197763

[B54] Zhang H. Bramham C. R. (2021). Arc/Arg3.1 function in long-term synaptic plasticity: Emerging mechanisms and unresolved issues. *Eur. J. Neurosci.* 54 6696–6712. 10.1111/ejn.14958 32888346

