# Supplementary material for: Estrous cycle state-dependent renewal of appetitive behavior recruits unique patterns of Arc mRNA in female rats
Source: Front Behav Neurosci. 2023 Jul 13;17:1210631. doi: 10.3389/fnbeh.2023.1210631 (PMC10372431; doi:10.3389/fnbeh.2023.1210631)
Supplement: Supplementary file 1 [file Data_Sheet_1.docx]

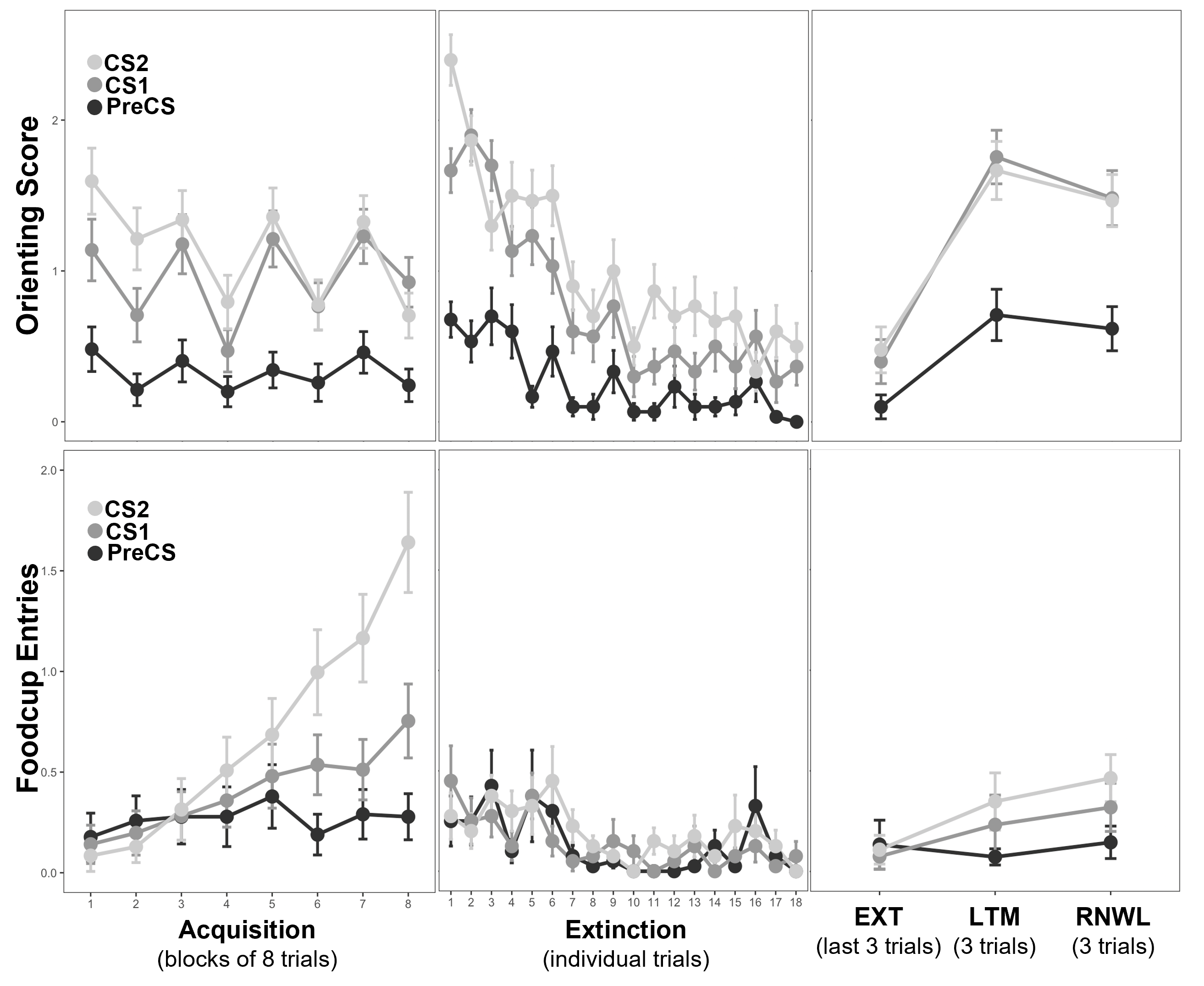


*sFig 1.* **(Top row)** Orienting responses (OR) +/- SEM and **(Bottom row)** Foodcup (FC) entries +/- SEM for acquisition (left), extinction training (middle), and testing (right). PreCS, 5s before the light CS illumination and CS1 and CS2, two blocks of 5s during the CS illumination.

*
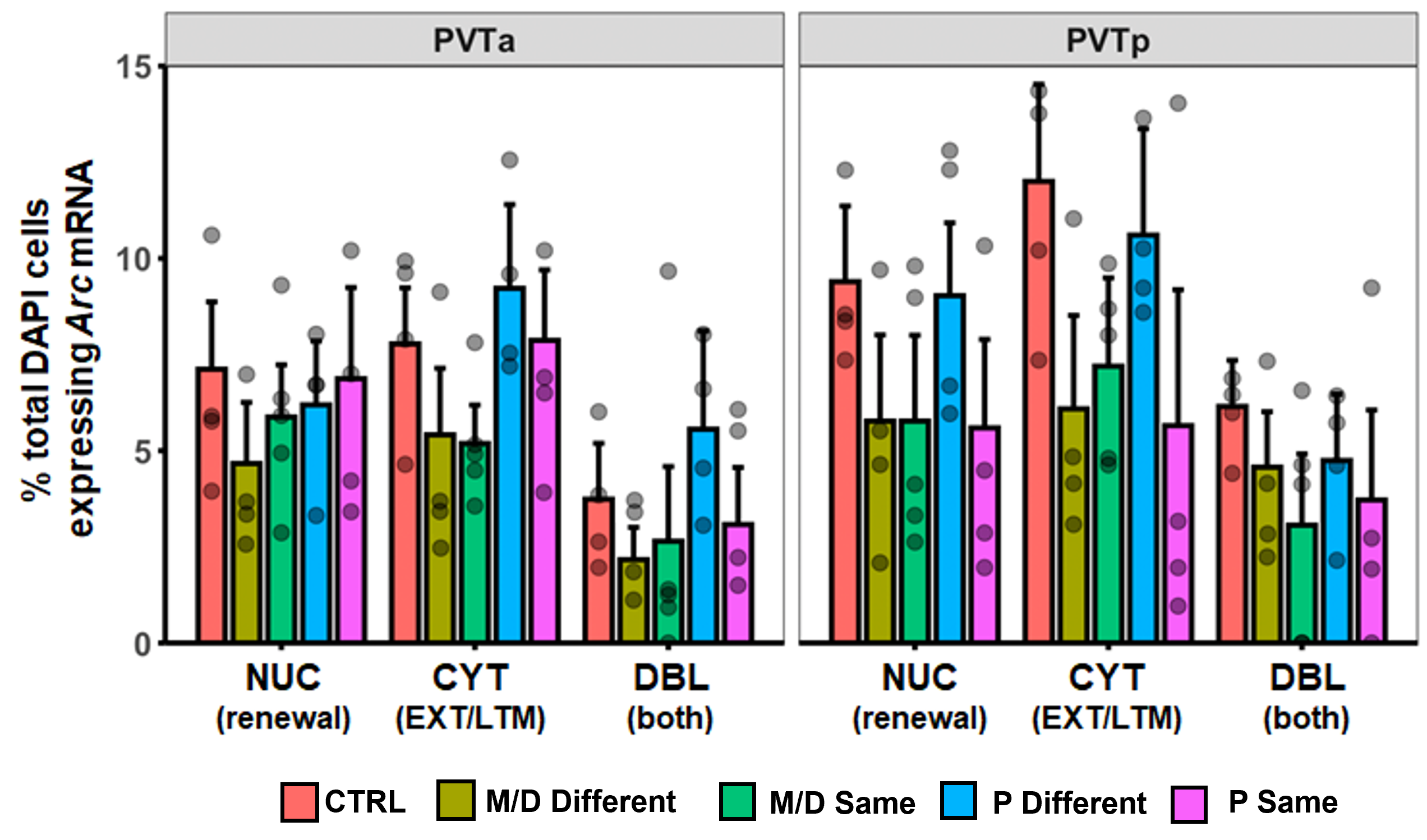
*

*sFig 2.* Percent of DAPI+ cells +/- SEM expressing CYT, DBL, or NUC *Arc* mRNA in PVTa (left) and PVTp (right).

*Arc* mRNA expression did not differ between groups in either region.
